# Functional analysis of the transition zone protein Rpgrip1l during zebrafish development

**DOI:** 10.1186/2046-2530-1-S1-P79

**Published:** 2012-11-16

**Authors:** C Vesque, A Mahuzier, I Anselme, M Leroux-Berger, SJ Schneider-Maunoury

**Affiliations:** 1 UMR 7622 CNRS, ERL INSERM 969, Université P. et M. Curie, France

## 

We investigated the function of Rpgrip1l during zebrafish development. Rpgrip1l encodes a protein localised at the ciliary transition zone and interacts functionally with NPH and MKS for the formation and function of the ciliary gate. The human *RPGRIP1L* gene is a causal gene in Meckel and Joubert type B syndromes, characterised by polydactyly, kidney cysts and central nervous system malformations. Using morpholinos injection, we show that loss of Rpgrip1l function leads to several early phenotypes such as convergent-extension phenotype, randomisation of left-right asymmetry and hydrocephaly. We focussed our study on the Wnt-PCP defects and we demonstrated that in the zebrafish floorplate, Rpgrip1l is required for correct positioning of the basal body along the planar polarity axis. We confirmed Rpgrip1l function on basal body positioning in the mechanosensory hair cells of the cochlea of the murine mutant for Rpgrip1l. Our results strongly suggest that Rpgrip1l is essential for recruiting and stabilizing dishevelled, a major actor of the PCP pathway, at the basal body and/or cilium. Indeed, in two different cell types, in the zebrafish floor plate and in the murine cochlea, dishevelled proteins are enriched at the cilium and/or basal body, and this localization is severely perturbed upon Rpgrip1l depletion. Finally, we demonstrate that, in the zebrafish floor plate, the function of Rpgrip1l in basal body positioning is mediated by dishevelled. We propose that Rpgrip1l participates in a protein complex required for recruiting and stabilizing dishevelled at the cilium, a process essential for planar polarization of the basal body.

